# Shared Storybook Reading and Oral Language Development: A Bioecological Perspective

**DOI:** 10.3389/fpsyg.2020.01818

**Published:** 2020-08-26

**Authors:** Lorenz Grolig

**Affiliations:** Max Planck Research Group Reading Education and Development (REaD), Max Planck Institute for Human Development, Berlin, Germany

**Keywords:** shared storybook reading, home literacy environment, language development, vocabulary, narrative, comprehension, ecological model, assessment

## Abstract

Shared reading research has become increasingly multidisciplinary and has incorporated a multitude of assessment methods. This calls for an interdisciplinary perspective on children’s shared reading experiences at home and at the child care center and their relationships to oral language development. Here, we first discuss Bronfenbrenner’s bioecological model of human development (Bronfenbrenner and Morris, 2006) regarding the relationship between shared storybook reading and oral language development. Second, we develop a framework for investigating effects of shared reading on language development in two important microsystems: the home literacy environment (HLE) and the child care literacy environment (CCLE). Zooming in on shared storybook reading as a proximal process that drives oral language development, we then develop a triad model of language learning through shared storybook reading that integrates approaches and evidence from educational psychology, developmental psychology, psycholinguistics, and corpus linguistics. Our model describes characteristics of children, adults, and books, and how their interplay influences shared reading activities. Third, we discuss implications for the Home Literacy Model (Sénéchal and LeFevre, 2002, 2014) regarding the conceptualization of shared reading as an important source of oral language development. Finally, to facilitate integrated research designs that include the two most important microsystems, we provide a critical discussion of assessment methods used in research that investigates the HLE and the CCLE and relate them to the shared reading triad in our bioecological model of shared storybook reading. We conclude with directions for future research.

## Introduction

Being proficient in the majority language is a key competence for learning in educational contexts, such as child care and school (Hoff, 2013; Kempert et al., 2019). Evidence from empirical developmental studies favors a usage-based theory of language acquisition (e.g., Tomasello, 2009) over theories postulating that language development is by and large an innate process (e.g., Chomsky, 1980). To become proficient speakers of a language, children need both communicative opportunities and proficient language models (Hoff, 2006). Longitudinal studies show marked differences in children’s vocabulary and grammar skills and in their rate of language acquisition as early as the first year of life, and these individual differences are strongly related to children’s language environments (Kidd et al., 2018).

Oral language comprehension is a major limiting factor in reading comprehension after children have acquired basic reading skills (i.e., fluent and accurate decoding of single words). Reading research differentiates between lower level language skills, which are related to word and sentence processing (e.g., vocabulary and grammar skills), and higher level language skills, which are related to the processing of texts (e.g., comprehension monitoring and narrative skills). The simple view of reading (Hoover and Gough, 1990) describes reading comprehension as the product of decoding and linguistic comprehension. Accordingly, both are necessary for understanding written texts. A child with poor decoding or oral language skills will most likely show poor reading comprehension. Oral language skills become increasingly important for reading comprehension in relation to word reading skills between Grades 1 and 4 (Storch and Whitehurst, 2002; Language and Reading Research Consortium, 2015a; Lervåg et al., 2018; Hjetland et al., 2019). This developmental trajectory has been found in relatively transparent orthographies, such as Spanish, Slovak, Czech (Caravolas et al., 2019), Finnish (Torppa et al., 2016), and German (Ennemoser et al., 2012).

Even though our understanding of reading acquisition has seen considerable progress in recent decades (e.g., Castles et al., 2018), there is still a substantial proportion of children who experience severe difficulties while learning to read. For example, results from the Progress in International Reading Literacy Study (PIRLS) 2016 study show that by the end of Grade 4, there are already large differences between high achievers and low achievers in Germany (Bos et al., 2017). At the end of Grade 4, about 19% of the school children in Germany have severe reading comprehension problems and need additional support to acquire adequate reading skills (Bos et al., 2017). Therefore, more research investigating the impact of early literacy environments on children’s language development is needed.

Previous reviews have synthesized evidence from shared reading research and developed models of environmental influences on language development (Fletcher and Reese, 2005; Hoff, 2006; Jaeger, 2016). In the present review, we take an interdisciplinary perspective on children’s shared storybook reading experiences at home and at the child care center and how they are related to the development of oral language and reading skills. To establish a theoretical framework, we discuss models of environmental influences on child development, focusing on Bronfenbrenner’s bioecological model with regard to language development during early childhood in section Environments and Language Development. We summarize evidence for the role of socio-cultural, educational, and familial factors for language development. In addition, we develop a framework for the effects of shared reading on language development in the home literacy environment (HLE) and the child care literacy environment (CCLE). In section Shared Reading in the HLE, we summarize evidence regarding concrete characteristics of the HLE that are related to the development of language and reading abilities. We develop a triad model of oral language learning through shared book reading that integrates approaches and evidence from educational psychology, developmental psychology, psycholinguistics, and corpus linguistics research. The model describes characteristics of children, adults, and books, and how their interplay influences shared reading activities. We propose modifications to the Home Literacy Model (Sénéchal and LeFevre, 2002, 2014) regarding the conceptualization of shared reading as an important source of language development, and the language outcomes are proposed. In section Assessment of Literacy Environments and Shared Reading, in order to facilitate integrated research designs that include the two most important microsystems, we provide a critical overview of assessment methods used in HLE and CCLE research, such as measures of socioeconomic status (SES), literacy environment questionnaires, behavior observations, diary methods, and recognition and recall tests. Finally, in section Summary and Directions for Future Research, we summarize the evidence for the triad model of shared reading and discuss avenues for future research.

## Environments and Language Development

In this section, we first summarize Bronfenbrenner’s bioecological framework for understanding human development. Afterwards, we take a look at shared reading and early literacy research through the lens of Bronfenbrenner’s framework and develop a bioecological model of oral language learning through shared reading.

### Bronfenbrenner’s Bioecological Model

Bronfenbrenner characterizes human development as a function of the interplay between psychological, biological, and environmental factors. The bioecological model of human development (Bronfenbrenner, 1977; Bronfenbrenner and Morris, 2006) describes different social spheres as the environmental contexts in which child development occurs. The interplay between a child and another person (e.g., family members, child care workers, and peers) is conceptualized as a microsystem, which is a “pattern of activities, social roles, and interpersonal relations experienced by the developing person in a given face-to-face setting with particular physical, social, and symbolic features that invite, permit, or inhibit, engagement in sustained, progressively more complex interaction with, and activity in, the immediate environment” (Bronfenbrenner, 1994, p. 1654). Microsystems influence the child’s development directly and are also reciprocally influenced by the child. Due to the direct engagement of children in microsystems, these environments are regarded as proximal influences on child development. The combination of and relationships between two or more interacting microsystems is called mesosystem (e.g., communicative practices at home and at the child care center).

An exosystem, by contrast, is conceptualized as distally influencing child development. It consists of connections and transmissions between two or more settings, of which at least one is not an immediate environment to the child (e.g., a parent’s workplace), and therefore, an exosystem can have indirect effects on a child’s development (e.g., a parent who works late spends less time interacting with the child in the evening). The exosystem includes, for example, characteristics of the parents’ workplace that affect the time parents spend with their children or regulations by educational institutions that affect preschool curricula. Finally, the most distal influence on child development is exerted by the macrosystem, which consists of cultural values, norms, and laws that can be specific for people of different social classes, religious confessions, or nationalities (Bronfenbrenner and Morris, 2006).

Earlier versions of the bioecological model have stressed the importance of investigating the influence of each system component on human development (e.g., Bronfenbrenner, 1977). Most of the ensuing research, however, has revealed that proximal processes in microsystems are the “primary engines of development” (Bronfenbrenner and Morris, 2006, p. 798), which has led to an intensified interest in these processes. Proximal processes are the interactions between a child and other persons in the child’s immediate external environment. Proximal processes need to operate regularly and over a sufficient time span to have an effect on the person’s development. The latest version of the bioecological model describes human development primarily as a function of a “progressively more complex reciprocal interaction between an active, evolving biopsychological human organism and the persons, objects, and symbols in its immediate external environment […]” (Bronfenbrenner and Morris, 2006, p. 797). Proximal processes that are developmentally effective include active involvement of the developing person and reciprocal interactions between people, objects, and symbols. Proximal processes develop in accordance with the developmental course of the involved persons. Over time, they become more complex to meet the developmental needs and to support further development of the persons.

To investigate environmental influences on development, research should take into account that the power of proximal processes (e.g., shared reading) depends both on the environmental context (e.g., shared reading at home and at the child care center) and characteristics of the person (e.g., memory; Bronfenbrenner and Morris, 2006). Interactions between environmental factors and person variables are of key interest in bioecological research: “The form, power, content, and direction of the proximal processes effecting development vary systematically as a joint function of the characteristics of the developing person and the environment – both immediate and more remote – in which the processes are taking place […]” (Bronfenbrenner and Morris, 2006, p. 798). Effects of proximal processes vary as a function of the developing person’s characteristics, most notably a child’s dispositions for engaging in proximal processes that can help to initiate and sustain proximal processes. For example, children who show an active interest in picture books are more likely to ask caregivers to be read to, and they might prefer this activity over other activities such as watching a series or physical activities. By contrast, children who find it in general hard to focus on the story of picture books are less likely to demand being read to, and they might prefer other activities over shared reading.

Additionally, personal resources are important developmental variables, such as ability, experience, and knowledge (Bronfenbrenner and Morris, 2006). For example, effects of shared reading on oral language skills might depend on children’s prior oral language skills, shared reading experiences, and knowledge about the contents of a picture book. In turn, developmental outcomes of these proximal processes (e.g., vocabulary and narrative skills that were facilitated through shared reading) are themselves resources that help to extend the effects of the proximal processes (e.g., more advanced extratextual talk between a child and the caregiver during shared reading that supports the development of higher level language skills). According to Bronfenbrenner and Morris (2006), bioecological research should focus on the specific aspects of the behaviors that are assumed to be most closely related to the developmental outcome, for example, investigating which aspects of literacy environments are most closely related to oral language development. Finally, effects of proximal processes also vary as a function of the more remote environmental contexts into which the proximal processes are embedded, the historical periods in which the proximal processes occur, and the developing person’s biological systems. The biological systems within a developing organism both limit individual development and represent at the same time the potential for development that can be realized through adequate experiences (Bronfenbrenner and Morris, 2006).

### A Bioecological Model of Language Development Through Shared Reading

**Figure 1:**
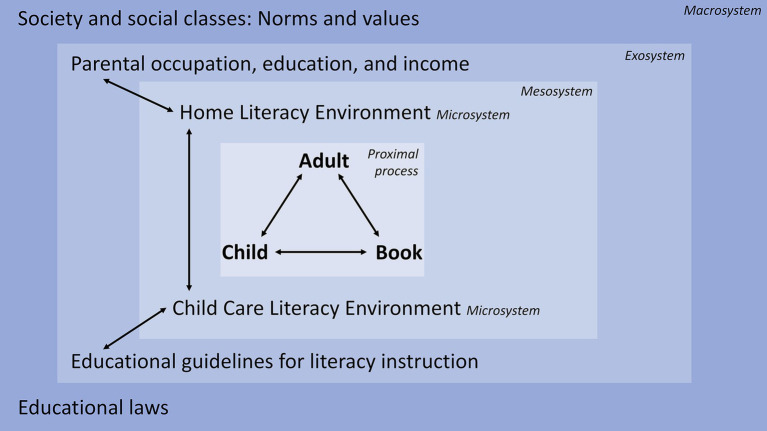
A bioecological model of oral language development through shared reading.

In this section, we develop a bioecological model of oral language development through shared reading (see Figure 1 for an overview of the components). Most children acquire sufficient oral language skills for everyday communication purposes, regardless of the amount or quality of shared reading they experience. Meta-analytic evidence shows that the correlation between oral language skills, such as vocabulary and grammar skills, and print exposure increases considerably between preschool (*r* = 0.34) and college (*r* = 0.66; Mol and Bus, 2011). Shared reading (and later independent reading) is not the only proximal process that fosters oral language development, but it is one main driving force behind oral language individual differences, and the most important source of variability in oral language skills that are precursors of reading comprehension (e.g., vocabulary; Montag et al., 2015).

The proximal process of shared reading can be described through relationships between child, adult, and book, which we describe in our triad model of oral language development through shared reading (see section Determinants of the Shared Reading Triad’s Effects on Language Skills). According to Vygotsky (1978), children can extend their language skills when they act in the zone of proximal development in collaboration with adults: Children’s learning is facilitated through a guided participation in culturally determined, meaningful situations. Children’s language skills are supported by adults’ input, questions, and feedback, which creates a “scaffold” that facilitates children’s development in the zone of proximal development, allowing them to reach a higher level of functioning. Repeated scaffolding enables children to internalize these more advanced modes of action and apply them independently in similar situations. Crucially, the influence of adults on the language development of children is mediated through the shared use of psychological and technical “tools” that are culturally shaped, such as children’s books. During the interaction, a conversation or activity is co-constructed between a child and another person (Vygotsky, 1978). Children can become more active in the co-construction of narratives and also understand the more difficult concepts if they are embedded in concepts that they have already mastered, and adults can discuss in a more sophisticated way about the story after a basic understanding of the story has been established (van Kleeck, 2003).

Regarding the model presented in Figure 1, several person variables can influence how children and caregivers interact during shared reading. Bronfenbrenner and Morris (2006) distinguish between resource characteristics (e.g., oral language skills, general cognitive skills), demand characteristics (e.g., literacy interest), and force characteristics (e.g., reading motivation). Moreover, shared genes and gene-by-environment interactions constrain the extent to which children’s oral language skills are malleable through environmental factors such as shared reading. Several studies found that the shared environment explains more variance in oral language skills than genetic differences in early childhood (Spinath et al., 2004; Chow et al., 2011; Olson et al., 2011; Hayiou-Thomas et al., 2012). One longitudinal study reported that oral language skills have a low heritability before school entry, but heritability increases between age 7 and 16 (Tosto et al., 2017). In conclusion, both genetic and shared environment influences constrain the maximum effect of early literacy interventions on young children’s oral language skills. A preliminary conclusion drawn from the limited empirical evidence is that aiming to support the development of oral language skills seems to be reasonable because their heritability at preschool age and subsequent years is lower than the heritability of decoding precursors, potentially benefitting reading comprehension in early primary school (Tosto et al., 2017). Finally, the proximal effects of shared reading on oral language skills also depend on book characteristics, such as the lexical and grammatical diversity of the text (Montag et al., 2015).

On the microsystem level, educational research has identified two environments that are related to children’s oral language development through shared reading: the HLE and the CCLE (Sénéchal et al., 1996; Weigel et al., 2005; Ebert et al., 2013; Niklas and Schneider, 2013; Weinert and Ebert, 2013). Some studies found that the HLE is more closely related to oral language than the CCLE (Ebert et al., 2013; Weinert and Ebert, 2013; Grolig et al., 2019), whereas other studies found that the influence of both literacy environments had a similar magnitude (Weigel et al., 2005; Schmerse et al., 2018).

On the mesosystem level, there are potential connections between HLE and CCLE, but few studies have investigated connections between the two (Weigel et al., 2005; Schmerse et al., 2018). In a large-scale German study, children’s vocabulary skills benefitted more from high child care language process quality if they experienced a medium or high quality HLE rather than a low quality HLE (Schmerse et al., 2018). By contrast, a U.S. study did not find that interactions between caregivers’ activities or beliefs in the HLE and CCLE predicted vocabulary skills or development (Weigel et al., 2005). Due to the limited number of studies, the magnitude and the source of concurrent and longitudinal environmental effects are unclear (see Hoff, 2006, for a review).

On the exosystem level, parents’ occupation, education, and income are important predictors of oral language skills at preschool age (Hoff, 2006). As they are highly interdependent, the three predictors are often combined to form a SES variable (Buckingham et al., 2014). Children from lower SES families are exposed to only about one-third of the oral language input quantity that children from higher SES families get (Hart and Risley, 1995). On average, kindergarten children from poor neighborhoods receive much less language input and less diverse language input with regard to vocabulary and grammar from their parents and teachers than children from lower middle class neighborhoods during shared reading, play situations, and classes, leading to slower growth rates in expressive vocabulary skills (Neuman et al., 2018). The language of parents with a lower SES often has a lower lexical diversity in comparison to the language of parents with a higher SES (Burchinal et al., 2008; Huttenlocher et al., 2010). As a consequence of these input differences, children with a higher SES background often have a larger vocabulary (Hart and Risley, 1995; Hoff, 2006; Gilkerson et al., 2017) and more often use diverse and advanced grammatical constructions than children from lower SES families (Huttenlocher et al., 2002). Importantly, communicative and shared reading practices not only vary between families as a function of their SES, but they can also differ considerably between families with a similar SES. Within groups of SES (lower vs. middle vs. upper SES), there is a large variability of communicative practices, such as the amount and linguistic characteristics of talk between parents and children and the frequency of shared book reading in the family (Hoff, 2006; van Steensel, 2006). Therefore, SES does not determine the amount and quality of literacy activities at home, even though children from lower SES households are on average more likely to receive less literacy activities than children from higher SES households. Another important influence on the exosystem level is educational guidelines for language education and language fostering in the child care center (e.g., Ruberg and Rothweiler, 2012) because they provide an orientation for effective oral language activities. For example, recent approaches to child care language education highlight the importance of the professional’s understanding of the general linguistic background of language development, the instrumental use of language as a key motivator for children, and the use of general communicative principles in everyday situations for implicit language teaching (Ruberg and Rothweiler, 2012).

On the macrosystem level, reading research and educational policies have influenced the norms and values connected to shared reading practices. In the last 50 years, research has accumulated a large body of evidence showing that shared reading in the first years of childhood is important for literacy development in general, and for oral language development in particular (Bus et al., 1995; Mol and Bus, 2011). At the same time, the main benefits that caregivers associate with children’s books in early child care have changed since the 1980s from social, emotional, play, and general cognitive skills to specific early literacy skills, such as vocabulary, grammar, and narrative skills (van Kleeck and Schuele, 2010). Concerning parents’ literacy activities, there are some SES differences regarding the attitudes, beliefs, and values connected to education in general and early literacy in specific, which become apparent in “characteristic modes of language use and interaction” (Hoff, 2006, p. 75). For example, compared to parents with a higher SES, parents with a lower SES tend to value the promotion of their children’s literacy development less (Kluczniok et al., 2013), tend to value reading to their preschool children less (DeBaryshe, 1995), and exhibit a lower interaction quality with their child during shared reading (e.g., asking less questions, larger proportion of parent talk in relation to child talk, and less verbal distancing; Lehrl et al., 2012).

Another important factor on the macrosystem level is that in many countries, educational laws make it an obligation for child care workers to document and foster language development, especially if the children’s native language is not the majority language (e.g., Senatsverwaltung für Bildung, Jugend und Wissenschaft, 2017). Professional associations and educational administrations encourage parents and child care workers to use children’s books as a means for promoting children’s emergent literacy skills (e.g., National Association for the Education of Young Children, 2009; Senatsverwaltung für Bildung, Jugend und Wissenschaft, 2014). As a consequence, shared reading is almost universally seen as a highly desirable activity for child development promotion in Western societies, and depriving children of shared reading experiences is therefore often described as a major disadvantage with respect to later success in school in the public discourse (e.g., Stiftung Lesen, 2018).

In sum, Bronfenbrenner’s bioecological model of development highlights that psychological and technical tools (e.g., language and books) are used for the co-construction of meaning between a caregiver and a child. Ideally, caregivers scaffold children’s processes of meaning-making by providing a developmentally appropriate context in which children can relate new language knowledge to prior language knowledge (zone of proximal development; Vygotsky, 1978), thereby refining their oral language skills. Bronfenbrenner and Morris (2006) posit a strong reciprocity of caregiver-child interactions, emphasizing the active involvement of young children in educational processes such as shared reading. Moreover, development is conceptualized as an outcome of interactions between environmental and person variables, whereby proximal processes that take place in microsystems are considered to be the main drivers of change. Applied to oral language development, shared reading as a proximal process depends on child, adult, and book characteristics, and relationships between these three literacy agents. Studies have identified the HLE and the CCLE as the two main environments that are directly related to oral language development through shared reading. In comparison, parental SES is a more distal variable with regard to language development, which is nevertheless related to differences in shared reading practices and the diversity of parent language, and ultimately, children’s oral language development.

## Shared Reading in the HLE

Several studies have investigated the components of the HLE that are related to oral language development. Section HLE Components and Relationships to Early Literacy Skills summarizes which components of the HLE can be distinguished, and which of them are related to different early literacy skills. In section Determinants of the Shared Reading Triad’s Effects on Language Skills, we summarize evidence for a triad model of shared reading that is proposed as a framework for more detailed investigations of shared reading as a proximal process. In section A Modified Home Literacy Model: Introducing the Shared Reading Triad, we propose a modified version of the Home Literacy Model (Sénéchal and LeFevre, 2002) that incorporates this shared reading triad, allowing a more detailed understanding of how interactions between child, adult, and book and their characteristics affect language development.

### HLE Components and Relationships to Early Literacy Skills

**Figure 2:**
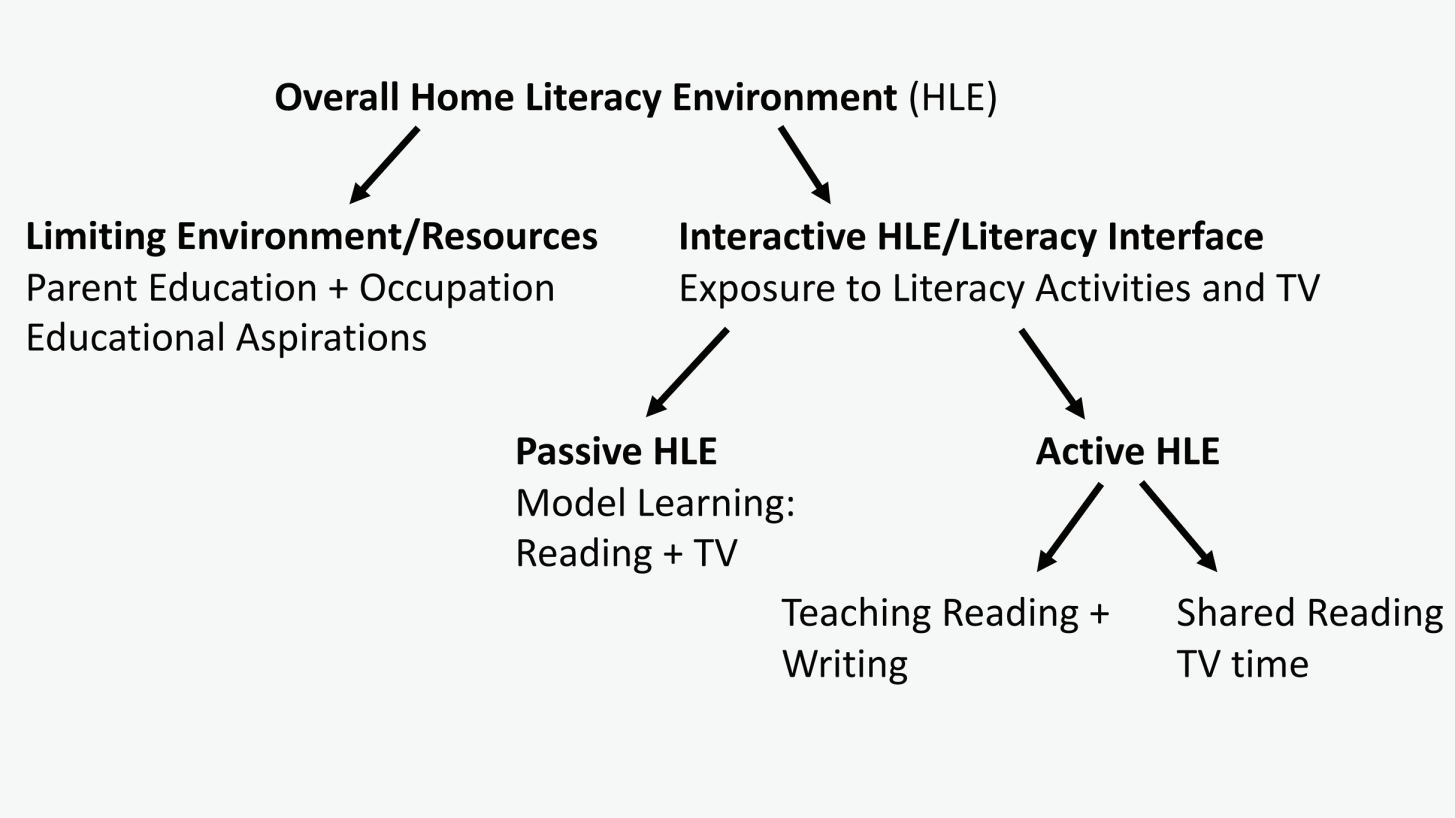
Components of the home literacy environment (HLE).

Components of the HLE can be divided into environment resources and exposure to literacy activities (see Figure 2). The latter includes passive HLE (model learning) and active HLE (shared reading, TV time). In addition, Sénéchal and LeFevre’s (2002) conceptualization of the HLE distinguishes formal teaching of writing and reading from shared storybook reading. Many studies have found that differences in the active HLE explain variance in early literacy and language skills over and above parent SES, literacy resources, and the passive HLE (e.g., Sénéchal et al., 1996; Burgess et al., 2002). This finding is consistent with the bioecological model of human development (Bronfenbrenner and Morris, 2006), positing that reciprocal interactions between active children and the persons and objects in their immediate environment are the main driving force of development. Therefore, more recent reading acquisition research has focused more on the active HLE than on the other components.

**Figure 3:**
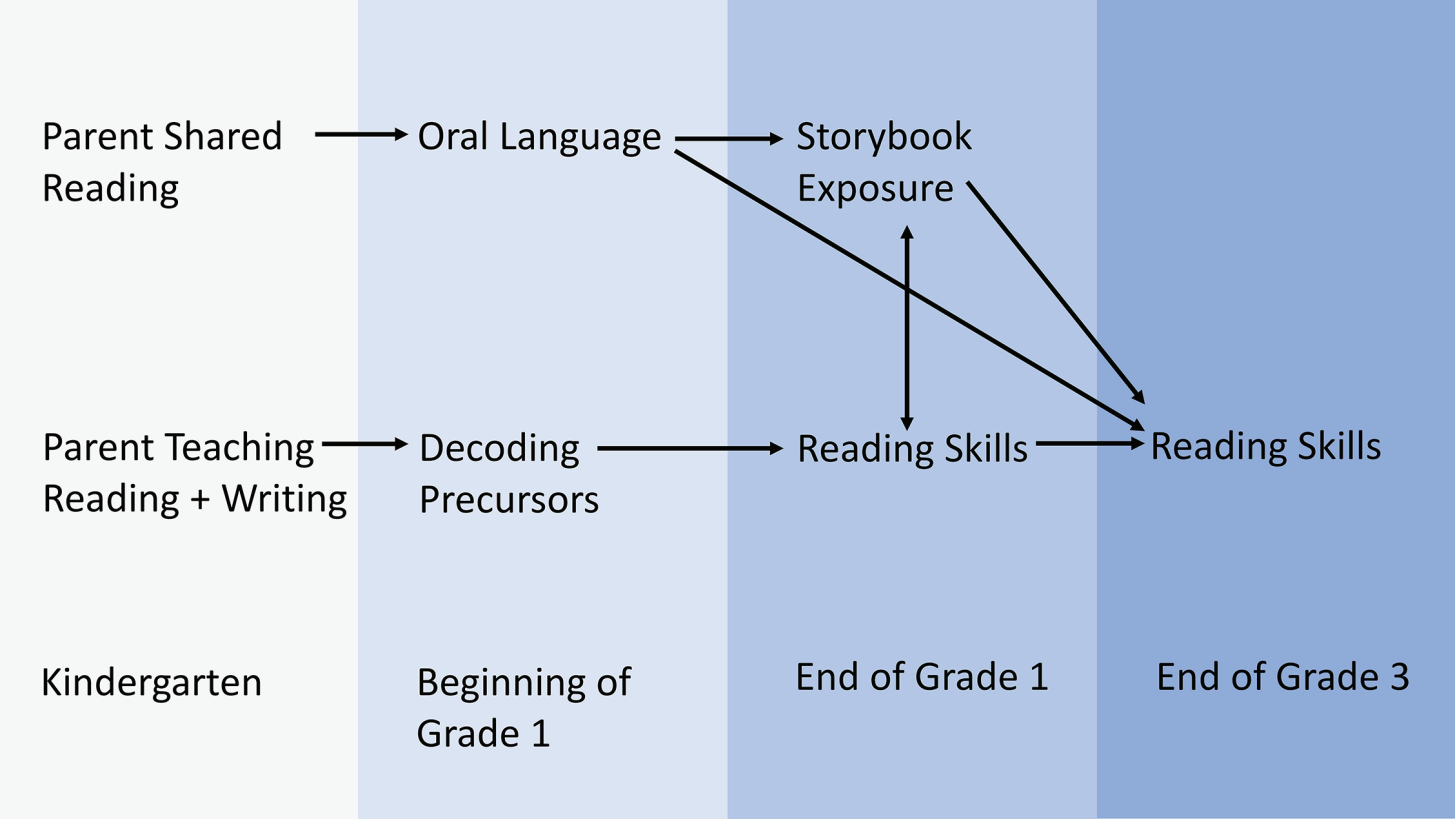
Home Literacy Model (adapted from Sénéchal and LeFevre, 2002). © 2002 by the Society for Research in Child Development, Inc. Adapted with permission.

The Home Literacy Model (Sénéchal and LeFevre, 2002, 2014 ^145^; see Figure 3) has been particularly influential. The model proposes that there are two independent parental influences that shape the HLE: Shared reading activities between parents and children, called informal HLE, support the development of oral language skills, such as vocabulary. By contrast, parental teaching of reading and writing skills, called the formal HLE, supports the development of decoding precursors, such as letter knowledge and phonological awareness.

The aim of the Home Literacy Model is to describe which specific parental activities and early literacy experiences support the acquisition of oral language skills and precursors of decoding skills in young children (Sénéchal and LeFevre, 2002). Evidence from longitudinal studies that were conducted in different cultures (e.g., Hood et al., 2008; Chen et al., 2010; Lehrl et al., 2013; Sénéchal and LeFevre, 2014) supports this proposed dichotomy. For example, a 5-year longitudinal study with English-speaking children found that informal and formal home literacy activities were not correlated, and that storybook exposure of kindergarten children predicted vocabulary development and comprehension skills at the beginning of Grade 1, which in turn predicted reading comprehension at the end of Grade 3 (Sénéchal and LeFevre, 2002). In the same study, parental teaching of reading and writing skills during kindergarten predicted precursors of decoding at the end of Grade 1, which in turn predicted reading comprehension in Grade 3.

Overall, the Home Literacy Model (Sénéchal and LeFevre, 2002, 2014) is a parsimonious evidence-based model. However, from a bioecological perspective, the model has several shortcomings. In particular, shared reading as a proximal process that drives oral language development seems to be underspecified. First, characteristics of child, adult, and book as literacy agents, their bivariate relationships, and their interplay should be taken into consideration. For example, motivation for leisure time reading in primary school declines during the first grades, exacerbating individual differences in reading skills (Wigfield et al., 2016). A more differentiated understanding of how children’s engagement during shared storybook reading can be enhanced could help to identify approaches for supporting reading motivation in primary school or even before. Second, even though different oral language skills on the word, sentence, and text level are highly correlated before school entry (Language and Reading Research Consortium, 2015b), there is some evidence that lower versus higher level language skills are each unique predictors of reading comprehension (Lepola et al., 2012; Kim, 2014; Catts et al., 2015; Silva and Cain, 2015). Therefore, a model of HLE’s effects on oral language should distinguish these two sets of language skills, and studies should investigate how they are related to shared reading.

### Determinants of the Shared Reading Triad’s Effects on Language Skills

On the level of shared reading as a proximal process of development (Bronfenbrenner and Morris, 2006), the communication during shared reading and its effects on oral language skills depend on the fit between the three literacy agents child, adult, and book (van Kleeck, 2003; Fletcher and Reese, 2005). Experimental and intervention studies investigating shared reading effects often observe that children only learn a fraction of the target words (Wasik et al., 2016). Many study designs are based on the manipulation of only a few shared reading variables and fail to mention other characteristics of the shared situation that are potentially important for secondary analyses (e.g., meta-analyses). To develop a better understanding of the interplay between these agents, it is helpful to consider the cognitive, motivational, emotional, and material characteristics that influence the shared reading process, including the specifics of the written language contained in children’s books. In addition to the characteristics of these three components, the relationships between them affect both the process and effectiveness of shared reading.

The inner rectangle in Figure 1 displays the triad model of shared reading in literacy environments in which adults, children, and books are involved in a proximal process that facilitates oral language development. This model is based on theoretical accounts of shared reading and literacy environments (van Kleeck, 2003; Fletcher and Reese, 2005; Jaeger, 2016) and evidence from empirical studies (see Hoff, 2006; Mol et al., 2008; Mol and Bus, 2011; Wasik et al., 2016; Flack et al., 2018, for reviews and meta-analyses). The main difference in comparison with previous models is a differentiation between characteristics of adults, children, and books involved in the shared reading process, their bivariate relationships, and the interplay of all three agents during shared reading. In the following, we discuss how characteristics of literacy agents and their relationships can affect shared reading.

#### Characteristics of Child, Adult, and Book

Theoretically, children’s language learning from shared reading should be related to differences in perceptive and cognitive functions that predict differential language learning from any environmental language input, such as phonetic distinction, wording segmentation from the speech stream, attentional functions (working memory and executive functions), and statistical learning (see Kidd et al., 2018, for a review). In a correlational study, the relationship between children’s storybook exposure and vocabulary skills was not moderated by verbal short-term memory, inhibitory control, or sustained attention (Davidse et al., 2011). In another correlational study, by contrast, working memory capacity moderated the relationship between HLE and language skills: The average language skills of children were lowest if they had a lower working memory capacity and came from a home that provided less shared reading activities (Leseman et al., 2007). Overall, evidence is scarce and inconclusive regarding the moderating role of children’s general cognitive functions with respect to language development. Moreover, there is a lack of research investigating whether effects of early literacy and language interventions are moderated by working memory or executive functions (Hasselhorn, 2010), which would allow causal inferences. The few studies that investigated differential effects of shared reading activities on language skills did not focus on such general cognitive functions but on verbal abilities (i.e., vocabulary) as moderator. Experimental studies found that children with higher pre-intervention vocabulary had larger language gains from shared reading (e.g., Sénéchal et al., 1995b; Coyne et al., 2009; Lenhart et al., 2019). Similarly, a meta-analysis of intervention studies found that dialogic reading with parents had very small effects on the oral language skills of children at risk for literacy and language impairments, whereas the effects on children not at risk were moderate (Mol et al., 2008).

Parents who believe that education and reading are important for child development provide shared reading activities to their children more often (DeBaryshe, 1995; Kluczniok et al., 2013). Additionally, parents who enjoy reading themselves are more likely to engage actively in shared reading with their children (Sonnenschein et al., 1997; Bus et al., 2000). Even more fundamentally, the language and reading skills of an adult, which depend to a large part on leisure time reading (Mol and Bus, 2011), are likely to determine the amount and quality of shared reading. For example, adults with low reading comprehension skills engage less frequently in shared reading activities with their children than adults with higher reading comprehension skills, presumably because reading is not an overly joyful leisure time activity to them (Neuman et al., 2018), and therefore, they are less likely to choose shared reading over other leisure time activities.

The characteristics of written language in children’s books are also important for explaining effects of shared reading on oral language skills. A children’s book can be analyzed as a “language model” (Hoff, 2006) that enables children to develop their language skills with the help of a reading person. On the word level, analyses of linguistic corpora have demonstrated that children’s books contain more diverse vocabulary than the language adults use in everyday situations with their children (called child-directed speech, CDS; Massaro, 2015; Montag et al., 2015). More specifically, the texts in books for children aged birth to 6 years contain more unique words, so-called types, than CDS of adults talking to children in the same age range (Montag et al., 2015). Moreover, children’s books contain a larger proportion of low frequency words (defined as words occurring less than 10 times per 1 million word tokens in a book corpus) than CDS in oral conversations (Crain-Thoreson et al., 2001; De Temple and Snow, 2003; Montag and MacDonald, 2015; Mesmer, 2016). Books present such words in semantic contexts that differ more than the semantic contexts of the CDS outside shared reading. Unlike most talk about the immediate environment, storybooks introduce words and concepts to the adult-child conversation that is independent from the situation in which the shared reading takes place (decontextualized language; Snow and Ninio, 1986; Nyhout and O’Neill, 2013). Being exposed to the same word in different contexts facilitates word learning and word recognition (Hills et al., 2010; Hsiao and Nation, 2018). As a consequence, shared reading not only facilitates the basic learning of new words (vocabulary breadth), but also the acquisition of the words’ semantic features (vocabulary depth; Ouellette, 2006). On the sentence level, corpus analyses have shown that children’s books contain more complex grammatical constructions than CDS (Cameron-Faulkner and Noble, 2013; Montag, 2019). Finally, on the text level, children’s books contain different narrative structures, providing a context in which children can learn to understand and (re-)produce narratives (Pantaleo and Sipe, 2012; Wagner, 2013, 2017).

#### Relationships Between Child, Adult, and Book During Shared Reading

The effects of shared reading on oral language development depend on the relationship and interaction between child and adult (Fletcher and Reese, 2005). Adults need to calibrate their communication to the child’s development in order to facilitate their learning in the zone of proximal development. More specifically, adults need to have a knowledge of a child’s language skills and prior world knowledge in order to select adequate books and ask questions of adequate difficulty. For example, the oral language skills of children with higher language scores benefit more from discussing stories than from the labeling and description of pictures, whereas children with lower language scores benefit more from the latter than from discussing stories (Reese and Cox, 1999; Zucker et al., 2010). In order to be effective, adults need to explicitly direct their talk during shared reading at the child (and maintain contact with the child) because talk that is not directed to children does not improve their oral language skills (Shneidman et al., 2013; Weisleder and Fernald, 2013).

Even before they become independent readers, children exhibit large differences in their interest in books, their motivation for shared reading, and their engagement during shared reading activities (Frijters et al., 2000; Hume et al., 2015). Studies have found that, while maternal reading behavior was not related to children’s engagement during shared reading, children’s engagement predicted language development and reading achievement (Crain-Thoreson and Dale, 1992; Dale et al., 1995). Similarly, the more questions children responded to during shared reading, the more words they learned (Sénéchal et al., 1995a; Sénéchal, 1997).

The relationship between adults and books is also an important factor in shared reading effectiveness. Adults differ in their preferences for reading over other leisure activities (Stanovich et al., 1995) and show large differences in print exposure (the amount of contact with written text; Stanovich and West, 1989). Moreover, adults with more print exposure exhibit better oral language skills (Mol and Bus, 2011), which are likely to influence their language use during shared reading. For example, while describing pictures, adults with more print exposure tend to use more complex grammatical constructions than adults with less print exposure (Montag and MacDonald, 2015). Parents often choose more complex books for shared reading with their preschool-aged children than for their younger children, reflecting that they are at least to some degree aware of their developmental differences (van Kleeck and Beckley-McCall, 2002).

#### Children’s and Caregivers’ Extratextual Talk During Shared Reading

The effects of some shared reading behaviors on language learning depend on the fit and the active coordination between all three literacy agents; for example, joint attention, extratextual talk, storybook selection, and repeated readings. One key question is how caregivers can facilitate children’s active engagement and language production during shared reading, and, in turn, their language learning.

The language production of adults and children in everyday situations is highly context-sensitive (Griffin and Ferreira, 2006; Dickinson et al., 2014). Children’s books allow the activation of a more diverse vocabulary than other communication settings because they provide very diverse language production contexts (Montag et al., 2015). For example, mothers’ talk during storybook shared reading with 5-year-old children contained more infrequent words (that were not included in the text of the book) than their talk during other activities (mealtime, toy play, magnet play, and information book reading; Weizman and Snow, 2001). The proportion of infrequent words was an important longitudinal predictor of children’s vocabulary in second grade (Weizman and Snow, 2001). In addition, several studies found that parents produce more grammatically complex sentences when reading a book with their children in comparison to their CDS while playing with their child. The mean length of parents’ utterances is longer, they respond more to the utterances of their children, and they use more abstract language (see Fletcher and Reese, 2005, for a review).

Language learning through shared reading is facilitated when adults and children engage in a sustained situation of joint attention (Ninio and Bruner, 1978; Fletcher et al., 2008; Farrant and Zubrick, 2013), which means that adults and children share a common (visual) focus with respect to a children’s book and that the two interact in this framework (e.g., pointing at and conversing about certain details of illustrations). For example, an experimental study found that instructing children to point at the illustrations of a children’s book during shared reading facilitates their word learning in comparison to passively listening to the adult’s reading (Sénéchal et al., 1995a). In addition, an intervention study found that caregiver contingent talk with infants facilitated their language production (McGillion et al., 2017). Other studies have found that infants can acquire a new object’s verbal label just by overhearing its name, which indicates that joint attention is not always necessary for some aspects of word learning (e.g., Gampe et al., 2012). Overhearing alone, however, is unlikely to be sufficient for acquiring a deep and nuanced comprehension of word meaning (i.e., vocabulary depth).

To establish joint attention, an adult activates and scaffolds a child’s thinking by (a) asking questions about a book’s contents (van Kleeck et al., 1997), such as asking the child to label depicted objects or asking to explain what happens on a certain page, (b) expanding the child’s answers, and which in turn (c) elicits new utterances from the child, and so on (dialogic cycle of communication during shared reading, Ninio and Bruner, 1978; Zevenbergen and Whitehurst, 2003). Many studies have found that asking basic comprehension questions during shared reading increases the effects on oral language skills in comparison to reading storybooks aloud without asking questions (see Wasik et al., 2016; Flack et al., 2018, for reviews). Asking such literal comprehension questions both serves to attain joint attention and to establish a fundamental understanding of concepts and events. Discussing the meanings of new words in the context of the story and in other contexts facilitates a deeper word understanding (Coyne et al., 2009).

Asking inferential comprehension questions in addition to literal comprehension questions can further enhance the positive effects of shared reading on vocabulary learning (Hindman et al., 2008; van Kleeck, 2008). Inferential questions also facilitated the production of narrative structures in two experimental studies (Silva et al., 2014; Silva and Cain, 2017), however, such a transfer effect was not found in an intervention study (Grolig et al., 2020a). Children’s books contain story grammar elements of which parents make use during shared reading: They produce story grammar elements that are contained both in the text and in the pictures of the books (Breit-Smith et al., 2017). Presumably, this exposure to story grammar elements and discussing them during shared reading helps children build an inner representation of story schemata, which in turn helps their understanding of oral and written stories (Fiorentino and Howe, 2004; Westerveld et al., 2008). Parents, however, rely heavily on contextualized utterances, that is, they stick closely to the literal textual and visual contents of books, focus often on the actions and only rarely combine this with more abstract contents such as inferences regarding figures inner states or plans (Breit-Smith et al., 2017). Even though inferential questions support the acquisition of higher level language skills such as narrative comprehension, parents generally ask more literal comprehension questions than inferential questions about the contents of a story (van Kleeck et al., 1997; Huebner and Meltzoff, 2005). How an adult and a child interact about a book depends on the interplay of all three literacy agents, such as (a) the adult’s propensity to ask open-ended questions during shared reading, (b) the child’s responsiveness to the adult’s questions and the contents in a storybook, and (c) features of the book that invite discussion, such as odd events.

The amount of pictorial information in relation to text-based information is also related to children’s engagement and the amount of extratextual talk. Using children’s books with illustrations during shared reading increases children’s engagement and parent-child extratextual talk compared to using matched books without illustrations (Greenhoot et al., 2014). In comparison to using children’s books with text during shared reading, using wordless picture books facilitates interactions between caregivers and children (Sénéchal et al., 1995a) and boosts the verbal production of both (Sénéchal et al., 1995a; Chaparro-Moreno et al., 2017). More specifically, in the study by Chaparro-Moreno et al. (2017), children produced more words (number of tokens), more diverse words (lexical diversity), and more sentences (number of utterances). At the same time, teachers produced more diverse words when using wordless picture books in comparison to storybooks with text. By contrast, the mean length of teachers’ utterances (sentences) was longer when using storybooks with texts compared to wordless picture books (Chaparro-Moreno et al., 2017), which is probably due to written sentences being longer and also more complex than spoken sentences in CDS (Cameron-Faulkner and Noble, 2013; Montag, 2019). Therefore, using wordless picture books instead of storybooks with text during dialogic reading is likely to be more effective in fostering vocabulary skills, but also likely to be less effective in fostering grammatical skills. Another study found that the amount and quality of mothers’ extratextual talk [i.e., lexical diversity and mean length of utterances (MLU)] does not differ when they read picture books with their children that contain more versus less text (Muhinyi and Hesketh, 2017), resulting in a doubled amount of extratextual talk during shared reading when using text-reduced children’s books, with no reduction in lexical diversity or mean length of utterances. Overall, evidence from these studies suggests that using wordless picture books during shared reading facilitates children’s oral language comprehension and production, with the exception of grammatical constructions that are typically found in written text.

Repeated readings of the same books can also increase children’s engagement (Morrow, 1988; Fletcher and Jean-Francois, 1998) and enhance their language learning through shared reading (Snow and Goldfield, 1983). Children who read a familiar book talk more than when reading a novel book (Fletcher and Reese, 2005). Moreover, parents and children talk more about related content or their own experiences when re-reading the same book, which also increases children’s world knowledge (Hayden and Fagan, 1987; Haden et al., 1996). For children with lower language abilities, repeated readings of the same book increase engagement in comparison to readings of different books (Morrow, 1988). Repeated readings provide multiple opportunities for repeated imitation (Ninio, 1983) and processing of novel words in a meaningful context (Sénéchal, 1997). Experimental studies have found that children’s expressive vocabulary is enhanced after two or more readings of the same book, whereas one reading often does not result in significant vocabulary gains (e.g., Sénéchal and Cornell, 1993; Sénéchal, 1997; Horst et al., 2011; McLeod and McDade, 2011).

### A Modified Home Literacy Model: Introducing the Shared Reading Triad

In sum, effects of shared reading on oral language are related to characteristics of children, adults, and books, such as (a) children’s prior oral language skills and presumably also their general cognitive functions, such as memory, (b) adults’ own reading habits and their beliefs about and attitudes toward shared reading, and (c) children’s books’ characteristics, such as lexical and grammatical diversity and narrative structures. Moreover, it is also important to consider bivariate relationships between children, adults, and books, because effects of shared reading on oral language skills depend on (d) adults’ ability to attract and sustain children’s attention and adjust their extratextual talk to children’s oral language skills level, (e) children’s interest in books and their engagement during shared reading, and (f) adults’ provision of children’s books at home, their ability to select developmentally appropriate books for shared reading with their children at different ages, and also their own print exposure, which is related to their oral language and reading skills. Finally, concerning the interplay of children, adults, and books, children’s engagement and language learning through shared reading can be enhanced by (g) establishing a common conversational focus with basic comprehension questions and (h) inferential comprehension questions during extended extratextual talk about vocabulary and story elements. Moreover, (i) repeated readings of (j) wordless picture books (or children’s books with relatively little text in comparison to pictures) facilitate children’s engagement and language production, and thus are effective means for increasing children’s oral language skills.

**Figure 4:**
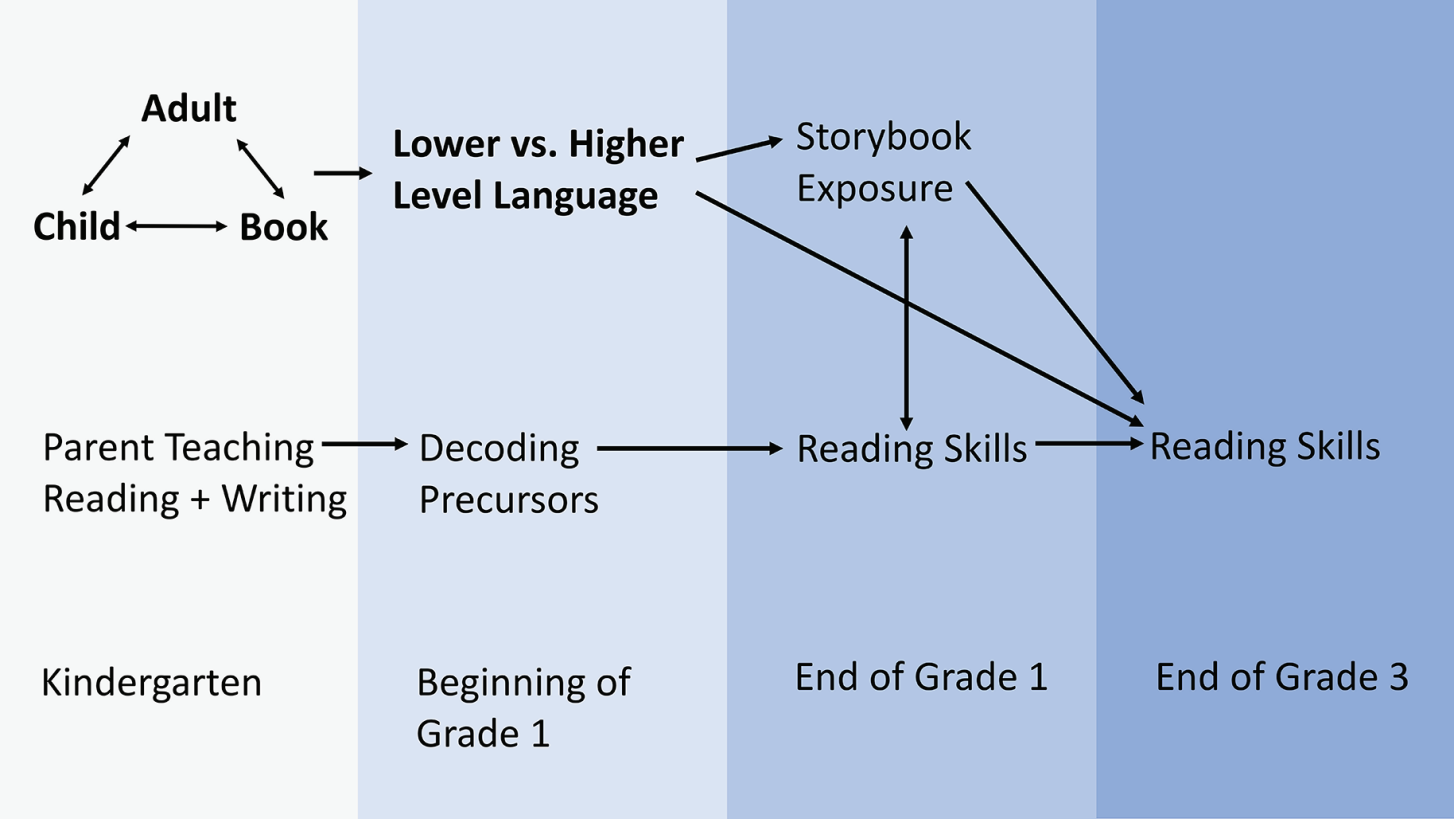
Modified Home Literacy Model with shared reading triad (adapted from Sénéchal and LeFevre, 2002; van Kleeck, 2003; Fletcher and Reese, 2005). © 2002 by the Society for Research in Child Development, Inc. Adapted with permission.

Based on the evidence summarized above, Figure 4 shows a modified model of the HLE. In comparison to the original HLE model (Sénéchal and LeFevre, 2002), the modified model (a) adds child and book as literacy agents to shared reading as a key activity before school entry that influences later oral language and reading development, (b) highlights the active role of children (cognitive, motivational, and socio-emotional variables), (c) highlights the role of book characteristics and book selection, incorporating evidence from corpus linguistics into a shared reading research framework, (d) differentiates between direct effects of literacy agents and the reciprocal influences between three literacy agents that also affect oral language development, and (e) differentiates between lower and higher level language skills as outcome measures of shared reading.

This modified model of the HLE conceptualizes shared reading as a complex process. In addition, shared reading as a proximal process is itself dynamic, changing over time in relation to children’s language, attention, and socio-emotional development, which is presumably related to changes in adults’ shared reading behaviors and characteristics of children’s books for different ages. This implies that key variables for the effects of shared reading on oral language skills need to be identified to allow a complexity reduction in empirical studies. Effects of shared reading appear to be small when measured over a few months (Mol et al., 2009, Noble et al., 2019), but substantial when measured over several years (DeBaryshe, 1993; Farrant and Zubrick, 2013). Ideally, then, assessment of shared reading practices should capture the effects of shared reading activities over a relatively long time period. Otherwise, shared reading effects are likely to be underestimated (Noble et al., 2019). The following section discusses how literacy environments and shared reading activities in the HLE and the CCLE can be measured.

## Assessment of Literacy Environments and Shared Reading

Investigating how shared reading in microsystems (HLE and CCLE) is related to oral language development in early childhood depends on the availability of adequate assessment methods. Pioneering correlational and longitudinal studies often had severe methodological shortcomings, among them measures with low reliability and social desirability bias (Lonigan, 1994). Since then, the field has developed and validated methods that capture different aspects of literacy environments and shared reading, which can be categorized as measures of (a) early literacy activities and shared reading input (e.g., literacy questionnaires and author recognition test (ART); section Measures of Literacy Environments), (b) the interactional quality during literacy activities and shared reading (e.g., environment rating scales and linguistic quality measures; section Interaction Measures of Shared Reading), and (c) memory outcomes of engaging in meaningful shared reading activities (e.g., recall of story details, recognition of storybook titles; section Outcome Measures of Shared Reading). Finally, we discuss which assessment methods are best suited for specific research questions and how they are related to environmental models of language learning (section Which Method for Which Research Question(s)?).

### Measures of Literacy Environments

As measures of the input provided for children through literacy environments and shared reading, studies have used SES, caregiver questionnaires, activity diaries, and the ART. In addition, linguistic approaches to oral language learning through shared reading have recently started to investigate the relationship between the lexical and grammatical input qualities of storybooks and children’s language development (e.g., Montag et al., 2015; von Lehmden et al., 2017; Wagner, 2017). In the future, this research will hopefully provide methods that are useful for the assessment of literacy environments and shared reading activities.

#### Socioeconomic Status

SES is a comparatively broad construct that is often operationalized as parent education, occupation, and income, or some combination of these variables (Buckingham et al., 2014). In the bioecological model (see Figure 1), it is situated on the exosystem level. Correlational and longitudinal studies corroborate that parent SES is positively associated with literacy activities (Fletcher and Reese, 2005; Hoff, 2006; van Steensel, 2006) as well as language and reading development during early childhood (Hart and Risley, 1995; Huttenlocher et al., 2002; Gilkerson et al., 2017). For example, parents with a middle SES report more shared reading than parents with a lower SES (Heath, 1983; Teale, 1986; Adams, 1990; Hammer, 2001; Britto et al., 2002). Whereas lower SES of parents is often associated with less frequent shared book reading, the effect of shared reading is not moderated by SES (Bus et al., 1995; Noble et al., 2019), indicating that children’s oral language skills benefit from shared reading regardless of their social background.

Measures of SES provide important information on the broader context in which children grow up. They are, however, less helpful in determining which specific activities are particularly effective in fostering language development (Lonigan, 1994). SES is a “catch-all” variable that is theoretically difficult to grasp because it includes many aspects that are shared with HLE activities and resources (e.g., number of books in a household), but also many additional aspects that are more generally related to child development (e.g., nutrition, healthcare, amount of stress experienced by parents and children, and time available for educational activities; Lonigan, 1994). In sum, SES is an important context variable for estimating the extent to which social inequalities are related to differences in language development. In educational research, it should be used in combination with indicators of proximal processes that provide specific insights into how oral language skills can be fostered.

#### Literacy Environment Questionnaires

Between the 1950s and 1990s, the informal HLE has most often been measured by single or multiple items in parent questionnaires, such as frequency of shared reading, the number of children’s books at home, parental leisure reading habits, family TV consumption, and frequency of family library visits (Bus et al., 1995). Meta-analyses have found that literacy activities (frequency of shared reading) and literacy resources (number of children’s books at home) are particularly robust predictors of language skills, and that questionnaire measures of the HLE explain about 8–12% of variance in children’s language skills (Bus et al., 1995; Mol and Bus, 2011).

Regarding the CCLE, few studies have used staff questionnaires to assess literacy activities and resources in the child-care setting (e.g., Weigel et al., 2005; Slot et al., 2015) and found that literacy activities in the CCLE were a unique predictor of vocabulary growth (Weigel et al., 2005). A meta-analysis found that domain-specific questionnaires did not explain a significant amount of variance in children’s outcomes (e.g., language and literacy skills), possibly due to a lack of reliable questionnaire measures available for the assessment of the quality of literacy activities in the CCLE (Ulferts et al., 2019).

In sum, questionnaires are valid and cost-effective proximal measures of literacy activities and resources in the HLE. There are, however, several disadvantages to them that limit their predictive power. First, at least in Western societies, norms and values prescribe that reading to children is important for their development, often resulting in social desirability bias when questionnaire measures are used. Parents tend to over-report literacy activities, thereby diminishing the usefulness of questionnaire measures for differentiating between children who experience more versus less shared reading activities (DeBaryshe, 1995). This can also constrain the variability of responses to questionnaire items and result in ceiling effects (e.g., Sénéchal et al., 1996; Davidse et al., 2011), reducing the magnitude of correlations between such questionnaire measures and language skills. Second, even if there is sufficient variability, questionnaire items can be still problematic when they ask for the average number of shared reading sessions or the average time spend with shared reading during a week. Due to memory constraints, most participants are not capable of providing reliable retrospective accounts of the average time they spend with different activities over periods of time (e.g., Bradburn et al., 1987; Burt and Kemp, 1991).

#### Activity Diaries

Activity diaries can be less prone to social desirability bias when participants are not informed that the research is specifically about leisure reading (Greaney, 1980). Participants fill in a form with a time grid for each day in which they describe everything they have done on this day (e.g., Smith, 2000; Ennemoser and Schneider, 2007). Activity diaries allow a more precise estimation of absolute reading times and rely less on participants’ memory abilities than questionnaire items that ask for retrospective estimation of average reading time. Even the duration estimation of recent events, however, is not immune to retrospection problems (Bradburn et al., 1987; Burt and Kemp, 1991). The main disadvantage of activity diaries is that they have to be filled in for several weeks to allow a generalization in terms of participants’ average leisure reading time. Therefore, diary measures require a high implementation effort, and participants need to be very motivated to comply over an extended period of time (Carp and Carp, 1981; Bolger et al., 2003).

#### Author Recognition Test

To circumvent social desirability and recall issues that come with literacy questionnaires and activity diaries, Keith Stanovich and colleagues developed a recognition test format that has been used with primary school children, adolescents, and adults (Stanovich and West, 1989; Cunningham and Stanovich, 1990; Allen et al., 1992). In the ART, participants indicate on checklists which names of bestselling authors they recognize. To discourage guessing, participants are informed that the list also contains fake authors (foils). To calculate a print exposure score that is corrected for guessing, the proportion of checked foils is subtracted from the proportion of checked real authors. ART scores are positively correlated with other measures of print exposure, such as reading habit questionnaires and activity diaries (Allen et al., 1992; see Mol and Bus, 2011, for a meta-analysis), real-world reading behaviors (West et al., 1993), and participant age (Grolig et al., 2020b). Moreover, adults’ ART scores also correlate positively with children’s and adults’ language and reading skills (West et al., 1993; Stanovich et al., 1995). Whereas activity diaries measure absolute reading times, recognition tests estimate relative differences in leisure reading time and related literacy activities.

In sum, the ART is a reliable, valid, and objective measure of print exposure that does not suffer from ceiling effects, social desirability bias, or imprecisions of event duration recall. With an administration time of about 5 min, the ART is also a very cost-effective measure. In early childhood research, parents’ scores in the ART are often used as a proxy of parental literacy (Sénéchal et al., 1996, 2008) or children’s print exposure (Puglisi et al., 2017). The main disadvantage of the ART is that the familiarity with author names differs between cultures. Therefore, the ART has been adapted for different cultures, including Chinese (Chen and Fang, 2015), Dutch (Brysbaert et al., 2020), German (Grolig et al., 2020b), and Korean (Lee et al., 2019). Also, the popularity of authors changes over comparatively short time spans. Therefore, the ART should be updated every 5 to 10 years for an optimal assessment of print exposure.

### Interaction Measures of Shared Reading

Whereas literacy environment questionnaires, activity diaries, and recognition tests focus on the quantity of shared reading, interaction measures also aim to assess quality features of literacy activities. In pedagogical research, observation measures are often used to characterize the quality of literacy-related interaction processes in the HLE and CCLE (section Observation Measures of Literacy Activities). Another approach to characterizing the quality of shared reading interactions is to analyze features of caregivers’ language during shared reading as predictors of children’s language development (section Linguistic Measures of Caregivers’ Speech and Extratextual Talk).

#### Observation Measures of Literacy Activities

Even though observation measures are considered to be less biased by social desirability than HLE questionnaires (Bus et al., 1995), few observation rating scales have to date been developed for the HLE that focus on early literacy activities or shared book reading in particular. For example, in a longitudinal large-scale study that tracked children’s development between age 3 and 10 in Germany (Pfost et al., 2013), a semi-standardized shared book reading task was used for rating the quality of the caregiver-child interaction (Family Rating Scale; Kuger et al., 2005; see Lehrl, 2018, for details). Raters assessed verbal distancing, nonverbal behavior, amount of (complex) questions, parent extratextual language, amount of children talk in relation to parent talk, and phonological cues (Lehrl, 2018). Interactional quality explained unique variance in grammar skills at age 3, but not in vocabulary skills. A brief HLE questionnaire (three items: quantity of books and children’s books in the household, shared reading frequency) explained unique variance in vocabulary and grammar skills at age 3 above the variance explained by the Family Rating Scale (Lehrl, 2018).

In educational research, standardized observation protocols and rating scales administered by external assessors are often used to characterize the quality of literacy-related interaction processes in the CCLE. Two of the most often used scales are the Early Childhood Environment Rating Scales (ECERS-R; Harms et al., 1998; ECERS-E; Sylva et al., 2003) and the Classroom Assessment Scoring System (CLASS; Pianta et al., 2008). Some of these scales, however, also assess structural aspects of early childhood education and care (ECEC) in addition to teacher-child interactions. Nevertheless, meta-analyses have reported positive correlations with children’s vocabulary skills. Both the ECERS-R total score and the language-reasoning subscale (using books and pictures, encouraging children to communicate, using language to develop reasoning skills, and informal use of language) are weakly related to the vocabulary skills of 30- to 72-month-old children (Brunsek et al., 2017). Moreover, the CLASS scale Instructional Support (concept development, quality of feedback, language modeling, literacy focus) is weakly correlated with vocabulary skills (Perlman et al., 2016). In addition, a meta-analysis of longitudinal studies found that environment rating scales that focus on the interaction quality and the observation of the process quality of domain-specific activities (e.g., language and literacy) result in relatively stronger correlations with vocabulary skills than scales that focus on the physical surroundings or questionnaire measures. The effect sizes, however, are in general small (Ulferts et al., 2019).

In sum, environment rating scales are reliable and valid direct measures of proximal processes that provide a detailed evaluation of the caregiver-child interaction. Scores are based on external raters which prevents bias due to social desirability. Considering that the literacy-related interactional quality in child care centers is often lower than desirable (Slot et al., 2015; Ulferts et al., 2019), environment rating scales are particularly useful for professional development interventions aiming to increase interactional quality (McNerney et al., 2006). On the other hand, the administration of environment rating scales is comparatively expensive because raters need to be trained for several hours, and on-site ratings often take two or more hours per classroom (e.g., Abreu-Lima et al., 2012). In addition, rating scales are not always significant predictors of preschoolers’ language skills (e.g., Powell et al., 2010; Hindman et al., 2012; Lehrl, 2018), possibly because the assessment is based on observations during one or 2 days, which might not be representative of the average quality of literacy activities in the CCLE (Slot et al., 2015). Interestingly, environment rating scales and questionnaires that aim to assess the same quality aspects of ECEC are only weakly correlated (Slot et al., 2015). Therefore, environment rating scales could be complemented by other measures that assess the average amount and quality of literacy activities over longer periods of time.

#### Linguistic Measures of Caregivers’ Speech and Extratextual Talk

Oral language development also depends on the quality of caregivers’ child-directed speech (CDS) and the extratextual talk associated with shared book reading. Linguistic measures of caregivers’ CDS, such as lexical diversity and mean length of utterances, are longitudinal predictors of preschoolers’ oral language development (Hoff-Ginsberg, 1998; Hoff and Naigles, 2002; Huttenlocher et al., 2010; Rowe, 2012; Weisleder and Fernald, 2013). These linguistic measures have also been used to investigate the effects of linguistic quality of extratextual talk.

In the HLE, parents use more low frequency words and complex sentences when they read a book with their children in comparison to other activities (e.g., Crain-Thoreson et al., 2001; Noble et al., 2019). In turn, the proportion of low frequency words and the syntactic complexity in parents’ extratextual talk during shared reading both predict preschoolers’ growth of vocabulary skills (Weizman and Snow, 2001; Baker et al., 2015). In the CCLE, the lexical diversity and syntactic complexity of caregivers’ CDS is also higher during shared book reading than during other activities (Dickinson et al., 2014). Similar to the findings in the HLE, the proportion of low frequency words (Dickinson and Porche, 2011) and complex syntax (Huttenlocher et al., 2002; Vasilyeva et al., 2006) in caregivers’ CDS predicts children’s growth in vocabulary and grammar skills.

In sum, deriving linguistic measures from observations of CDS is a valid and objective method for assessing literacy environments and shared reading activities. Similar to environment rating scales that provide detailed information about caregiver-child interactions on a behavioral level, linguistic measures provide a characterization of interactional quality features in educational settings that aim to foster oral language development (see Rowe and Snow, 2019, for a review that discusses linguistic, interactional, and conceptual dimensions of language input). Therefore, evidence from linguistic measures can be used for the development of preschool curricula, and also for professional development feedback. Linguistic measures, however, often cannot be derived automatically from recorded speech (see Gilkerson et al., 2017, as an example of automated analysis). More often, the audio material is manually coded, requiring many hours of work by trained staff. Therefore, linguistic measures are comparatively expensive.

### Outcome Measures of Shared Reading

By adopting the rationale behind the ART (Stanovich and West, 1989), early childhood researchers have developed specific recognition and recall tests for the assessment of young children’s storybook exposure. Whereas the ART is an input measure of literacy environments, storybook recognition and recall tests are outcome measures of shared reading activities. They assess relative differences in the recall of details from popular storybooks (section Storybook Knowledge Recall Tests) and the recognition of popular storybooks’ titles (section Storybook Title Recognition Tests). Storybook information is memorized and retained as a result of shared reading activities that are meaningful to children.

#### Storybook Knowledge Recall Tests

Children are asked to name a book’s title after they have seen its title page. If a title is correctly recalled, children are asked to tell some of the story details in order to control for guessing (Sénéchal et al., 1996; Davidse et al., 2011; Zhang et al., 2018). The recall scores explain a substantial amount of unique variance in children’s vocabulary skills after controlling for the broader HLE and background variables (Sénéchal et al., 1996; Davidse et al., 2011; Zhang et al., 2018).

Storybook knowledge recall tests are objective and valid measures of print exposure. The administration time depends on the number of book covers presented to children. This test format, however, is rarely used, presumably because it has disadvantages that reduce its explanatory power. Most notably, a successful recall of book title and story details poses high demands on children’s cognitive skills, which could explain the floor effects often found in these measures (Sénéchal et al., 1996; Davidse et al., 2011). Also, confounds with memory, attention, and language skills are problematic in studies investigating the relationship between shared reading and oral language skills.

#### Storybook Title Recognition Tests

Storybook title recognition tests (TRTs) are often used for examining the relationship between shared reading activities in the HLE and children’s language development (see Mol and Bus, 2011, for a meta-analysis). TRTs are most often administered as paper and pencil tests in which parents mark the storybook titles that they recognize (e.g., Hood et al., 2008; Hamilton et al., 2016) but can also be administered as audio decision tests to preschoolers (Grolig et al., 2017). As in the ART, the proportion of checked foils is subtracted from the proportion of checked real titles, resulting in a hit rate that is corrected for guessing. Parents’ TRT score is moderately correlated with HLE questionnaire measures and is considered to be a proxy of children’s print exposure (Mol and Bus, 2011).

In sum, storybook TRTs are objective, reliable, and valid measures of shared reading activities in the HLE that are less confounded with children’s cognitive skills than storybook knowledge recall tests. The test administration of the TRT takes about 5 min. The TRT has been adapted for many cultures in the last decades (e.g., Australian: Hood et al., 2008; Chinese: Ho, 2014; English: Hamilton, 2013; German: Grolig et al., 2017). The main disadvantage of the TRT is that the popularity of storybooks changes over time. Therefore, the storybook titles in the TRT need to be updated every 5–10 years for an optimal assessment of storybook exposure.

### Which Method for Which Research Question(s)?

Overall, there is no single method that fits all research questions. Each method has strengths and shortcomings. Therefore, combining measures with complementing strengths is the most reasonable approach to a comprehensive assessment of environmental influences on oral language learning. To understand how effects of shared storybook reading on oral language development are situated in communication settings, a comprehensive assessment of environmental factors should take into account distal environmental variables that are situated on the exosystem level (e.g., SES), proximal environmental variables that are situated on the microsystem level (e.g., descriptions of literacy environments), and descriptions or results of the proximal process itself, such as interaction or outcome measures of shared storybook reading.

**Figure 5:**
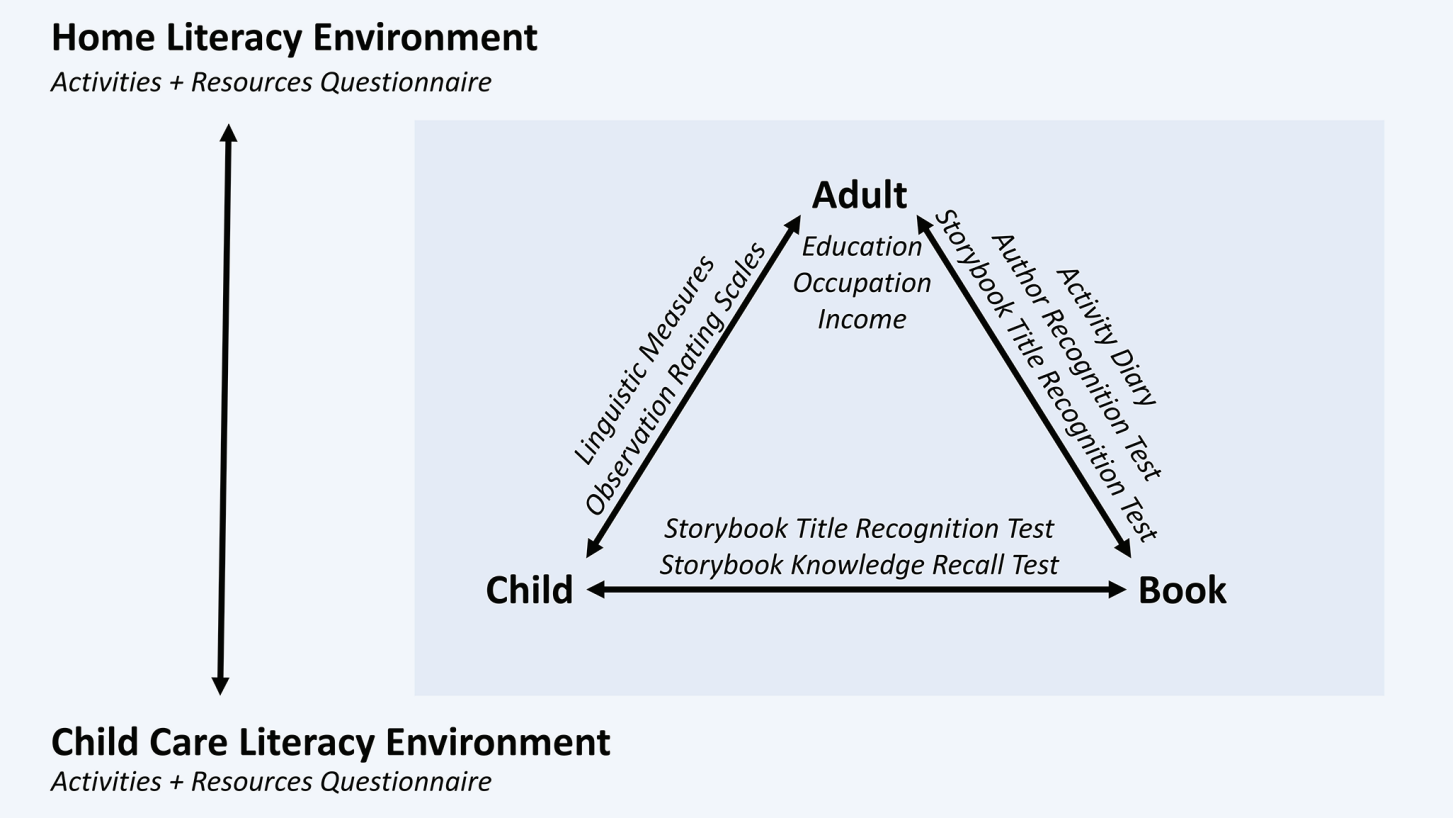
Measures for the assessment of literacy environments and shared book reading.

In general, the measures that were discussed in this section show an adequate dispersion of scores, with the exception of storybook knowledge recall tests, where floor effects can be problematic. In addition, the reliability of the measures is in general adequate or good, with the exception of staff questionnaires for the CCLE, where the reliability for some measures is relatively low (Ulferts et al., 2019). Figure 5 summarizes measures for the assessment of literacy environments and shared storybook reading and locates them in the shared reading triad of the modified home literacy model that was developed in section Determinants of the Shared Reading Triad’s Effects on Language Skills.

Considering that the influence of both HLE and CCLE on oral language should be assessed in sufficiently large samples to provide robust evidence for a bioecological model of language learning through shared reading, the amount of administration time and implementation effort are also critical factors that have to be considered. Most of the measures are relatively brief and cheap to implement; however, interactional measures (environment rating scales and linguistic measures) and activity diaries are much more time-intensive for researchers and participants, respectively. Therefore, environment rating scales and linguistic measures are probably used best when the evaluation of the interactional quality during shared reading or providing feedback during interventions is the focus of a study. Activity diaries provide the most reliable estimate of absolute leisure reading time, and therefore should be used in studies that investigate this specific variable.

Questionnaires about the HLE and CCLE are cost-effective measures for assessing the quantity of shared reading activities and resources. They also provide some basic description of shared reading activities and the physical environment but they often do not cover qualitative aspects. In addition, caregivers are aware that reading with children is beneficial for their development, which makes it more likely that they overstate the amount of shared reading. Besides this social desirability bias, items often ask for average occurrences of activities over an extended period of time, which leads to biases due to common event recall problems. Recognition test scores use foils as an effective control measure for social desirability. Also, they are based on the recognition of authors or titles, which is a simple memory process in comparison to averaging occurrences of shared reading over an extended time period, and therefore should be less confounded with memory abilities than questionnaire measures. Finally, recognition test scores reflect both long-term habits of leisure reading and recent reading activities because they contain classic and new authors (or storybook titles), capturing relative differences in shared reading activities over several years. Therefore, a cost-effective estimation of the relationships between the amount of shared reading in the HLE and the CCLE microsystems and language skills can be achieved by combining questionnaires and recognition tests.

## Summary and Directions For Future Research

Based on Bronfenbrenner’s bioecological model of human development (Bronfenbrenner and Morris, 2006), we developed a bioecological perspective on oral language learning through shared storybook reading and described how available research methods can be utilized to investigate key relationships between child, caregiver, and book, called the shared reading triad. In this review, we have integrated findings from psychological, educational, and linguistics research into an interdisciplinary bioecological framework that allows a comprehensive investigation of shared reading effects. We focused specifically on shared reading as a complex and changing proximal process that is a main driver of individual differences in oral language development.

Evidence from a large number of studies supports our triad model of shared storybook reading. This model can serve both as a research framework and provide some guidance for practitioners. First, effects of shared reading on oral language are related to characteristics of literacy agents, most notably children’s prior language skills, adults’ reading habits, and motivation toward shared reading, and children’s books’ lexical and grammatical diversity and narrative structures. Second, effects of shared reading are also related to relationships between these literacy agents. According to correlational and experimental studies, shared reading effects depend on children’s literacy interest and engagement as well as joint attention and adjusting extratextual talk to children’s oral language skills level. Moreover, regarding the relationship between adults and books, shared reading effects also depend on the provision of children’s books at home, adults’ ability to select developmentally appropriate storybooks, and also their leisure reading. Third, shared reading effects depend on the interplay of literacy agents. To get children engaged in shared storybook reading and activate their thinking, adults can use basic and inferential comprehension questions. Language learning is also facilitated by repeated readings and the use of wordless picture books because both increase children’s engagement and language production during shared reading. Another important outcome of this review is an evidence-based, modified Home Literacy Model that adds child and book as literacy agents, thereby highlighting the active involvement of children and book characteristics. The modified model differentiates both between direct effects of literacy agents and the reciprocal connections between them and between lower and higher level language skills as outcome measures. Finally, our discussion of assessment methods revealed that the combination of literacy environment questionnaires and recognition tests allows a cost-effective and sufficiently descriptive evaluation of long-term shared reading practices in literacy environments when qualitative aspects of shared reading interactions are not in the focus of the research.

Throughout this review, we have pointed out gaps in shared storybook reading research. The HLE and the CCLE are important microsystems in which children are likely to experience shared reading in a regular basis. Studies about their relative effects on oral language development and interactions between the two microsystems could inform practice and policy in order to support the development of children who come from disadvantaged families. More specifically, future studies of shared reading should aim to disentangle contributions of child, adult, and book plus their bivariate relationships and their interplay regarding effects on oral language skills. In particular, the moderating role of individual differences in language processing and gene by environment interactions need to be studied in detail. On these grounds, the magnitude of language education effects can be estimated, and individually tailored interventions could be developed. Presumably, text characteristics of storybooks contribute to oral language learning. Storybooks designed for experimental purposes will help to shed more light on this topic. Finally, children’s active engagement and language production during shared reading appear to be of key importance to language learning. In correlational and experimental research, a comprehensive description of the shared reading situation is needed to understand the contributions of these factors and their relationships, especially regarding secondary analyses. The triad model of shared storybook reading could help to establish a unified framework for shared storybook reading research.

## Author Contributions

LG contributed conception and design of the study. LG wrote the manuscript and revised the manuscript.

### Conflict of Interest

The author declares that the research was conducted in the absence of any commercial or financial relationships that could be construed as a potential conflict of interest.
